# Current drug use and lack of HIV virologic suppression: point-of-care urine drug screen versus self-report

**DOI:** 10.1186/1471-2334-14-508

**Published:** 2014-09-18

**Authors:** Han-Zhu Qian, Valerie J Mitchell, Sally Bebawy, Holly Cassell, Gina Perez, Catherine C McGowan, Timothy R Sterling, Sten H Vermund, Richard D’Aquila, Todd Hulgan

**Affiliations:** Vanderbilt Institute for Global Health, Vanderbilt University, Nashville, Tennessee USA; Division of Epidemiology, Department of Medicine, Vanderbilt University, Nashville, Tennessee USA; Division of Infectious Diseases, Department of Medicine, Vanderbilt University, Nashville, Tennessee USA; Vanderbilt Comprehensive Care Clinic, Nashville, Tennessee USA; Division of Infectious Diseases, Department of Medicine, Northwestern University Feinberg School of Medicine, Chicago, Illinois USA

**Keywords:** Drug use, HIV, Computer-assisted self-interview, Urine drug screen, Antiretroviral therapy, Virologic suppression

## Abstract

**Background:**

There have been inconsistent findings on the association between current drug use and HIV disease progression and virologic suppression. Drug use was often measured using self-report of historical use. Objective measurement of current drug use is preferred.

**Methods:**

In this cross-sectional study, we assessed drug use through Computer-Assisted Self Interviews (CASI) and point-of-care urine drug screen (UDS) among 225 HIV-infected patients, and evaluated the association between current drug use and virologic suppression.

**Results:**

About half (54%) of participants had a positive UDS, with a lower self-reported rate by CASI (42%) (*Kappa* score = 0.59). By UDS, 36.0% were positive for marijuana, 25.8% for cocaine, 7.6% for opiates, and 2.2% for methamphetamine or amphetamine. Factors associated with virologic suppression (plasma HIV RNA <50 copies/mL) were Caucasian race (*P* = 0.03), higher CD4 count (*P* < 0.01), current use of antiretroviral therapy (ART) (*P* < 0.01), and a negative UDS (*P* < 0.01). Among 178 current ART users, a positive UDS remained significantly associated with lower likelihood of virologic suppression (*P* = 0.04).

**Conclusions:**

UDS had good agreement with CASI in detecting frequently used drugs such as marijuana and cocaine. UDS at routine clinic visits may provide “real-time” prognostic information to optimize management.

## Background

The introduction of antiretroviral therapy (ART) has resulted in a decline in AIDS-defining events and mortality in HIV-infected populations. Studies suggest, however, that people who use drugs (PWUD), particularly those who inject drugs, may not achieve the same benefits from treatment as persons who do not use drugs [1, 2]. Even when controlled for adherence to ART, outcomes for PWUD may be poorer compared to non-users [3]. However, other studies have shown conflicting results with minimal or no association between drug use and HIV disease progression [4, 5]. These discordant findings may be due to differences in measuring drug use, such as the type of drugs used, route of administration, dose and frequency, current or past use, and polydrug use [4, 6].

There are numerous ways of measuring drug use in research and clinical care settings. Traditional paper-based questionnaire surveys, either self-completed or administered by an interviewer, are relatively inexpensive and easy to implement, but are subject to social desirability bias due to stigma associated with drug using behaviors [7, 8]. Computer Assisted Self Interview (CASI) may reduce but not eliminate such bias [9–11]. Both questionnaire and CASI interviews can assess past and current use of drugs. In contrast, direct drug testing by urine drug screen (UDS) detects metabolites in urine samples with good sensitivity and specificity [12, 13], and therefore provides an objective assessment, but is more costly and may be impractical for routine use in some settings. As the window for detecting metabolites in urine typically ranges from a few hours to two weeks, a positive UDS indicates current or recent drug use [13]. Studies on the relationship between use of illicit drugs and HIV disease outcomes often use self-reported history of drug use. There is a scarcity of literature on validation of reported drug use and on the assessments of current drug use and its potential association with HIV treatment outcomes among HIV-infected patients [3, 14]. We assessed the agreement between CASI and UDS results in this group of HIV-infected participants, and the relationship between virologic suppression and drug use as assessed by UDS.

## Methods

### Study setting and target population

The study was conducted during June 2010 and December 2011 at the Vanderbilt Comprehensive Care Clinic (VCCC), a large outpatient HIV clinic in Nashville, Tennessee that provides integrated HIV and psychiatric care, and social work services to over 2,800 patients in active care. An estimated 80% of patients at VCCC are from Middle Tennessee; the remaining are from other parts of Tennessee or from surrounding states of Kentucky, Georgia, West Virginia, Alabama and Arkansas. Patients were recruited by a research nurse during clinic visits at VCCC, where they read brochures about this drug use-related study then voluntarily decided to participate in the study. About one out of three patients approached by the nurse agreed to participate in the study. Each participant provided a written informed consent for completing a CASI interview, providing urine sample for UDS, and giving permission of use of their clinical data. A $10 gift card was given to participants who completed the study procedures. The study protocol was approved by the institutional review board of Vanderbilt University. Approval was granted to incorporate data collected from this study into the VCCC data and specimen repository for future research use, but study data were kept separate from the medical record.

### Computer-assisted self interview

CASI was conducted to collect data on drug and alcohol use, depression, and adherence to antiretroviral therapy (ART). Participants were asked 3-item AUDIT alcohol consumption questions (AUDIT-C) to query for problematic drinking [15, 16]. Participants were also asked to indicate whether or not they had used a variety of illicit substances in the past 7 days, 6 months and lifetime using a modified version of WHO-ASSIST (Alcohol, Smoking & Substance Involvement Screening Test) questionnaire. Substances included cocaine/crack, marijuana, heroin or other opiates, methamphetamine (crystal meth), pain medications, benzodiazepines, and inhalants. The 9-item Patient Health Questionnaire (PHQ-9) Depression Module was used to screen for depression. The 4-item AIDS Clinical Trials Group (ACTG) Questionnaire for Adherence to Antiretroviral Medications was used to assess ART adherence [17].

### Urine drug screen

All participants undertook a point-of-care UDS. The FDA-approved *QuickScreen™* Pro 5 Drug Test Card (Craig Medical Distribution, Inc. Vista, CA) was used [18]. It is a one-step panel immunoassay for the qualitative detection of amphetamine, benzoylecgonine (cocaine metabolite), methamphetamine (including Ecstasy), opiates (morphine/heroin metabolite) and marijuana in human urine. Amphetamine has a half-life of 4–24 hours in the body and methamphetamine has a half-life of 9–24 hours; cocaine can generally be detected for 24–48 hours after exposure; the half-life of opiate drugs may range from 2 to 32 hours, e.g., 8–12 hours for heroin; marijuana remains detectable for 3–10 days after smoking. The visual one-step panel urine test provides a qualitative “yes or no” report in 5 minutes. According to the package inserts of the testing kit, the agreement rates of detecting the individual drugs using the *QuickScreen™* Pro 5 Drug Test Card and confirmatory gas chromatography–mass spectrometry range from 95% to 98%. A trained research nurse performed UDS and recorded the results. While waiting for UDS results, patients completed CASI.

### Clinical data

Data retrieved from the clinical database at VCCC included socio-demographics, ART use, and the latest values of CD4 count and HIV RNA in plasma measured by standard procedures as part of routine care. The values of CD4 and viral load that were closest to the survey date were used in the analysis, most within 3 months of the survey and few between 3–6 months. Virologic suppression was defined as plasma HIV RNA <50 copies/mL.

### Data analysis

The AUDIT-C is scored on a scale of 0 to 12, and the scores of ≥4 in men and ≥3 in women were defined as unhealthy alcohol use [15, 19]. Current drug use was defined as a positive result on any of five tested drugs in UDS. We did not assess relationships with specific drugs due to limited study sample size. The PHQ-9 is scored on a scale of 0 to 27, and the scores of ≥10 were defined as major depression (mediate or severe depression) [20]. ART adherence was defined by whether having missed at least one dose of ART in past 4 days. Virologic suppression was defined as HIV-1 RNA < 50 copies/mL.

We calculated the detection rate of drug use by UDS and the rates of drug use during the past 7 days, the past 6 months, and lifetime use by CASI self-report. We then calculated the agreement (*Kappa* score) of detecting current drug use by CASI 7-day report and UDS. We considered self-reported 7-day drug use as current use. The *Kappa* scores are interpreted as: < 0 indicating no agreement, 0–0.20 slight, 0.21–0.40 fair, 0.41–0.60 moderate, 0.61–0.80 substantial, and 0.81–1 almost perfect agreement [21].

We performed multivariate logistic regression analysis in the entire study sample to evaluate the relationship between current use of any drugs as determined by UDS and virologic suppression while adjusting for covariates. We also performed subgroup analysis among patients who had received ART only, because prescription of ART may be less likely to occur for drug using patients than for non-users [22]. We selected covariates and fitted regression models based on our cohort analysis in the same study population [1]. We re-analyzed the data and did not find interaction between ART and drug use. Therefore, we use the parsimonious models without interaction term.

## Results

A total of 226 HIV-infected patients participated in the study; one participant did not provide a urine sample for UDS and was excluded from the analysis. Of 225 participants, about 30% were female; the mean age was 43 years (median 49; range 19–66); 38% were Caucasians, and 59% were African Americans (Table 1).

**Table 1:** **Description of study participants by UDS status**

Variable	Total sample (N = 225)	UDS positive (N = 121)	UDS negative (N = 104)	*P*-value
Sex (male)	157 (69.8%)	86 (54.8%)	71 (45.2%)	0.65
Age, year (mean ± SD)	42.7 ± 10.1	44.0 ± 10.0	41.2 ± 10.2	0.04
Race				0.01
White	86 (38.2%)	40 (46.5%)	46 (53.5%)	
Black	132 (58.7%)	80 (60.6%)	52 (39.4%)	
Other	7 (3.1%)	1 (14.3%)	6 (85.7%)	
Unhealthy alcohol use	85 (37.7%)	49 (57.6%)	36 (42.4%)	0.71
Major depression (≥moderate level)	57 (25.3%)	29 (50.9%)	28 (49.1%)	0.67
ART, yes	179 (79.6%)	94 (52.5%)	85 (47.5%)	0.63

**Note:** UDS: urine drug screen; SD: standard deviation.

Nearly 38% of participants reported unhealthy alcohol use; 25% had moderate or severe depression (Table 1). Of the 80% of patients who were prescribed ART, 32% reported missing at least one dose in the past 4 days (data not shown).

Table 2 presents the prevalence of drug use by UDS and CASI. About half (53.5%) had a positive UDS for any drug. Over one third (36.0%) were positive for marijuana, 25.8% for cocaine, 7.6% of opiates, 2.2% for methamphetamine or amphetamine. The CASI reported rate of using any drugs in the past 7 days was 42.0%, which was significantly lower than that by UDS (less than the lower bound of 95% confidence interval of UDS rate). CASI also reported lower rates of marijuana (28.6%) and cocaine (18.9%) use than corresponding positive UDS rates. Ninety-two percent of participants reported any lifetime drug use. Though nearly a quarter of participants reported ever injecting drugs, very few reported injection drug use in the past seven days (0.4%) or six months (1.8%).

**Table 2:** **Prevalence of drug use by point-of-care urine drug screen (UDS) and Computer-Assisted Self Interview (CASI) among 225 HIV-infected patients in middle Tennessee, USA (%)**

Drug	UDS proportion (95% CI)*	CASI		
		Past 7 days	Past 6 months	Lifetime
Any drugs	53.5 (47.0-60.0)	42.0^‡^	59.3	92.4
Marijuana	36.0 (29.7-42.3)	28.6^‡^	44.1	85.0
Cocaine	25.8 (20.1-31.5)	18.9^‡^	29.5	66.8
Opiates	7.6 (4.1-10.1)	7.5	18.5	43.8
Meth/amphetamine	2.2 (0.3-4.1)	1.8	6.2	25.2
Injection	N/A	0.4	1.8	23.7

**Note:** CI: confidence interval; N/A: not applicable.

*95% CI was calculated in order to assess whether or not the CASI point estimate was likely to be within 95% CI of the UDS estimate.

^‡^The prevalence of 7-day drug use by CASI is less than the lower bound of 95% CI of the prevalence by UDS.

Table 3 presents the agreement statistic *Kappa* values for detecting drug use by UDS and CASI reported 7-day drug use. The *Kappa* values for marijuana and cocaine were 0.68 and 0.71, respectively, indicating substantial agreement; and the *Kappa* value for any drugs was 0.59, indicating moderate agreement. For other infrequently used drugs, the agreement was poor.

**Table 3:** **Agreement in detecting drug use by point-of-care urine drug screen (UDS) and Computer-Assisted Self Interview (CASI)**

CASI reported 7-day use		UDS			*Kappa*coefficient
		(+)	(−)	total	
Any drugs	(+)	84 (37.3%)	10 (4.4%)	94 (41.8%)	0.59
	(−)	37 (16.4%)	94 (41.8%)	131 (58.2%)	
	Total	121 (53.8%)	104 (46.2%)	225 (100.0%)	
Marijuana	(+)	57 (25.3%)	8 (3.6%)	65 (28.9%)	0.68
	(−)	24 (10.7%)	136 (60.4%)	160 (71.1%)	
	Total	81 (36.0%)	144 (64.0%)	225 (100.0%)	
Cocaine	(+)	39 (17.3%)	3 (1.3%)	42 (18.7%)	0.71
	(−)	19 (8.4%)	164 (72.9%)	183 (81.3%)	
	Total	58 (25.8%)	167 (74.2%)	225 (100.0%)	
Opiates	(+)	2 (0.9%)	15 (6.7%)	17 (7.6%)	0.05
	(−)	15 (6.7%)	193 (85.8%)	208 (92.4%)	
	Total	17 (7.6%)	208 (92.4%)	225 (100.0%)	
Methamphetamine	(+)	0 (0.0%)	4 (1.8%)	4 (1.8%)	−0.01
	(−)	5 (2.2%)	216 (96.0%)	221 (98.2%)	
	Total	5 (2.2%)	220 (97.8%)	225 (100.0%)	

**Note:** CI: confidence interval; (+): positive screen or reported use; (−): negative screen or reported no use.

Factors associated with virologic suppression were Caucasian race (versus non-Caucasian; adjusted odds ratio [AOR] 2.5; 95% confidence interval [CI] 1.1-5.7), CD4 count (per 10 cell/uL increase; AOR 1.003; 95% CI 1.001-1.004), current use of ART (AOR 8.3; 95% CI 3.1-22.3), and current drug use by UDS (a positive UDS was associated with a significantly lower odds of virologic suppression: AOR 0.3; 95% CI 0.1-0.7). There was still a statistically significant association between current drug use and lack of virologic suppression among the subgroup of participants who were currently prescribed ART (AOR 0.4; 95% CI 0.2-0.9) (Table 4).

**Table 4:** **Multivariate logistic regression analyses of factors associated with HIV suppression (<50 copies/mL) among 225 HIV-infected patients**

Variable	Model 1: overall			Model 2: ART users		
	(N = 225)			(N = 178)		
	Adjusted OR	95% CI	*P*-value	Adjusted OR	95% CI	*P*-value
Sex (male vs. female)	1.38	0.63-3.02	0.42	1.29	0.53-3.18	0.57
Age, 1 year increase	1.01	0.97-1.05	0.54	1.02	0.97-1.07	0.33
Race (Caucasian vs. non-Caucasian)	2.50	1.10-5.68	0.03	3.29	1.20-9.06	0.02
CD4 count, 10 cells/uL increase	1.003	1.001-1.004	<0.01	1.003	1.001-1.004	<0.01
Current use of ART	8.29	3.08-22.30	<0.01			
Missed at least one dose of ART in past 4 days				0.93	0.37-2.35	0.87
Current drug use by UDS	0.33	0.15-0.73	<0.01	0.38	0.15-0.94	0.04
Unhealthy alcohol use	1.12	0.52-2.44	0.77	1.02	0.43-2.44	0.97
Major depression (≥moderate level)	0.72	0.31-1.69	0.44	0.89	0.32-2.43	0.81

OR: odds ratio; CI: confidence interval; UDS: urine drug screen.

## Discussion

Our study is one of few studies comparing self-report and UDS in measuring drug use [14, 23–25] and the only study to our knowledge among HIV-infected patients. We found that CASI and point-of-care UDS had fair agreement in detecting current use of common drugs such as marijuana and cocaine among HIV-infected patients, but had poor agreement for infrequently used drugs. CASI tends to underreport current drug use compared to UDS. Only about 10% of participants (n = 24) ever received drug or alcohol treatment in the past 6 months, and the treatment experience had no significant impact on the difference of detecting drug use by UDS and self-report. A study among college students showed that the overall result in detecting any drug use was satisfactory, while the sensitivity of self-report on more stigmatized drugs such as cocaine was lower than that on less stigmatized drugs such as marijuana (cannabis) among college students [14], and these findings are consistent with another study among men who have sex with men and the general population [24]. One advantage of CASI is that it can assess drug use for longer periods of time, such as past 6 months or lifetime. However, serial UDS at repeated clinic visits may also allow longer-term assessment and is also less likely to be subject to reporting bias.

Lifetime and current drug use was very common among this group of HIV-infected, predominantly ART-treated participants recruited during clinical visits and thus engaged in care to some degree. Current drug use was associated with lower likelihood of virologic suppression among ART-treated participants. Drug use may lead to suboptimal HIV treatment outcomes through both biological and behavioral mechanisms. For example, PWUD may have poor adherence to ART; illicit drugs such as amphetamines, cocaine, marijuana, and opiates may alter immune function and increase susceptibility to infection [4, 26]. Rapid point-of-care screening for drug use at routine clinic visits may provide useful “real time” prognostic information for HIV treatment efficacy and optimize management decisions by identifying persons at greatest risk for treatment failure, guiding decisions regarding targeted adherence counseling, timely referral to appropriate drug use treatment programs, and testing for viral resistance. We did not find an association between unhealthy alcohol use and virologic suppression, although there is biological plausibility [27]. Evidences on the relationship between heavy alcohol consumption and HIV outcomes are inconsistent [27].

Our study suggests the potential treatment benefits of integrating routine screening for substance use in HIV clinics, with and without the use of UDS. The detection of drug use will alert physicians to consider providing drug abuse treatment and counseling in order to achieve optimal HIV treatment outcome. Meanwhile, there are numerous issues that may arise from implementing routine point-of-care UDS for all HIV-infected patients. Patients may have concerns about drug use status being disclosed to their employers or insurers, and their views on integrated HIV primary care and drug screening and treatment should be taken into consideration [28]. Physicians may provide differential care to drug-using patients, including deferring ART treatment [29]. The cost of UDS may not be reimbursed from patients’ insurance plans. Concerns about routine UDS may increase the likelihood that some patients would miss medical appointments. Therefore, instead of universal UDS for all HIV-infected patients, opt-out UDS could be considered. Finally, from the perspective of clinic administrators, implementing routine point-of-care UDS would require additional personnel and lab space.

Our study has several limitations. The length of time during which different drugs or their metabolites can be detected in urine samples depends on many factors, including chemical properties (e.g., half-life), metabolism rates and excretion routes, amount consumed, administration route, frequency and chronicity of use, and individual variations in patients’ physical health, exercise, diet, weight, gender, and fluid intake that may affect excretion rates [30], and it is generally 2–5 days with a range from 24 hours up to 14 days. The QuickScreen™ Pro 5 Drug Test Card is convenient to use but provides only a preliminary analytical test result; in comparison, confirmatory methods such as gas chromatography/mass spectrometry (GC/MS) are expensive. Therefore, our UDS findings might underestimate drug use. We did not assess use of prescription opioid use, which might be common among HIV-infected patients [31]. This topic warrants research. The study participants were recruited as a convenience sample through brochures advertising a drug use-related study. This recruitment likely led to an enrichment of the population who currently used drugs and an overestimation of current drug use, but it should not affect the validity of assessing test agreement or the relationship between current drug use and virologic suppression. Adherence to ART, a key covariate in assessing the relationship between drug use and viral suppression, was based on self-report, which may result in overestimation of adherence; pharmacy data and Medication Event Monitoring System (MEMS) could be considered as alternative measures [32, 33]. Due to the cross-sectional nature of the study, we could not ascertain the temporal relationship between drug use and virologic suppression. Prospective cohort studies with longitudinal assessment of point-of-care drug use screening and HIV outcomes are needed.

## Conclusions

UDS at routine clinic visits may provide “real-time” prognostic information to optimize HIV care, but studies are needed to evaluate its acceptability among patients and providers.
